# Baseline global longitudinal strain predictive of anthracycline-induced cardiotoxicity

**DOI:** 10.1186/s40959-021-00090-2

**Published:** 2021-01-31

**Authors:** Raquel Araujo-Gutierrez, Kalyan R. Chitturi, Jiaqiong Xu, Yuanchen Wang, Elizabeth Kinder, Alpana Senapati, L. Bindu Chebrolu, Mahwash Kassi, Barry H. Trachtenberg

**Affiliations:** 1 Houston Methodist DeBakey Heart and Vascular Center, 6550 Fannin St., Suite 1901, Houston, Texas 77030 USA; 2 Department of Medicine, Division of Cardiovascular Medicine, University of Missouri-Columbia, Columbia, Missouri USA; 3 Center for Outcomes Research, Houston Methodist Research Institute, Houston, Texas USA

**Keywords:** Global longitudinal strain, echocardiography, speckle tracking, anthracycline, cardiomyopathy

## Abstract

**Background:**

Cancer therapy-related cardiac dysfunction (CTRD) is a major source of morbidity and mortality in long-term cancer survivors. Decreased GLS predicts decreased left ventricular ejection fraction (LVEF) in patients receiving anthracyclines, but knowledge regarding the clinical utility of baseline GLS in patients at low-risk of (CTRD) is limited.

**Objectives:**

The purpose of this study was to investigate whether baseline echocardiographic assessment of global longitudinal strain (GLS) before treatment with anthracyclines is predictive of (CTRD) in a broad cohort of patients with normal baseline LVEF.

**Methods:**

Study participants comprised 188 patients at a single institution who underwent baseline 2-dimensional (2D) speckle-tracking echocardiography before treatment with anthracyclines and at least one follow-up echocardiogram 3 months after chemotherapy initiation. Patients with a baseline LVEF <55% were excluded from the analysis. The primary endpoint, (CTRD), was defined as an absolute decline in LVEF > 10% from baseline and an overall reduced LVEF <50%. Potential and known risk factors were evaluated using univariable and multivariable Cox proportional hazards regression analysis.

**Results:**

Twenty-three patients (12.23%) developed (CTRD). Among patients with (CTRD), the mean GLS was -17.51% ± 2.77%. The optimal cutoff point for (CTRD) was -18.05%. The sensitivity was 0.70 and specificity was 0.70. The area under ROC curve was 0.70. After adjustment for cardiovascular and cancer therapy related risk factors, GLS or decreased baseline GLS ≥-18% was predictive of (CTRD) (adjusted hazards ratio 1.17, 95% confidence interval 1.00, 1.36; *p* = 0.044 for GLS, or hazards ratio 3.54; 95% confidence interval 1.34, 9.35; *p* = 0.011 for decreased GLS), along with history of tobacco use, pre-chemotherapy systolic blood pressure, and cumulative anthracycline dose.

**Conclusions:**

Baseline GLS or decreased baseline GLS was predictive of (CTRD) before anthracycline treatment in a cohort of cancer patients with a normal baseline LVEF. This data supports the implementation of strain-protocol echocardiography in cardio-oncology practice for identifying and monitoring patients who are at elevated risk of (CTRD).

## Introduction

Routinely used in the treatment of hematologic malignancies and solid tumors, anthracyclines can lead to cancer therapy-related cardiac dysfunction (CTRD) in approximately 9% of patients and is mostly diagnosed within the first year of treatment in patients who are monitored prospectively. Known risk factors include high cumulative dose (i.e. doxorubicin ≥250 mg/m^2^) or any dose combined with radiation therapy in the heart field, sequential use of HER 2? her-2 antagonists, age> 60 years, cardiovascular risk factors, and compromised cardiac function (e.g. LVEF<55%, pre-existing ischemic or valvular disease) [1].

However, certain patients lacking these high-risk features develop anthracycline-induced cardiomyopathy. Earlier detection of subclinical myocardial dysfunction may identify these individuals, allowing for closer clinical monitoring during and after anthracycline therapy and prior to initiation of potentially cardioprotective interventions.

Two-dimensional speckle tracking echocardiography (STE) with global longitudinal strain (GLS) has become a well-established and important tool to predict subsequent CTRD (commonly defined as a decreased left ventricular ejection fraction (LVEF) <50 or 53% with ≥5% absolute reduction in symptomatic patients or ≥10% in asymptomatic patients) *during* anthracycline therapy, even in patients without risk factors for CTRD [2–6]. Recent retrospective studies of patients with hematological malignancies show that *baseline* GLS before initiation of anthracycline therapy is predictive of left ventricular systolic dysfunction or major adverse cardiac events, including cardiac death or symptomatic heart failure [7–9]. However, these studies included patients with borderline LVEF (50–55%). Our study objective was to evaluate the clinical utility of STE and baseline GLS in detecting CTRD in a *broad cohort* of cancer patients with a *normal* LVEF.

## Methods

The study was conducted at Houston Methodist Hospital with patients selected from the Houston Methodist Oncologic Pharmacy Registry, which includes cancer-related therapy information from the Houston Methodist Cancer Center and 7 hospitals and affiliated cancer centers. The institutional review board of Houston Methodist approved the protocol.

### Study population and pre-specified risk factors

**Fig. 1:**
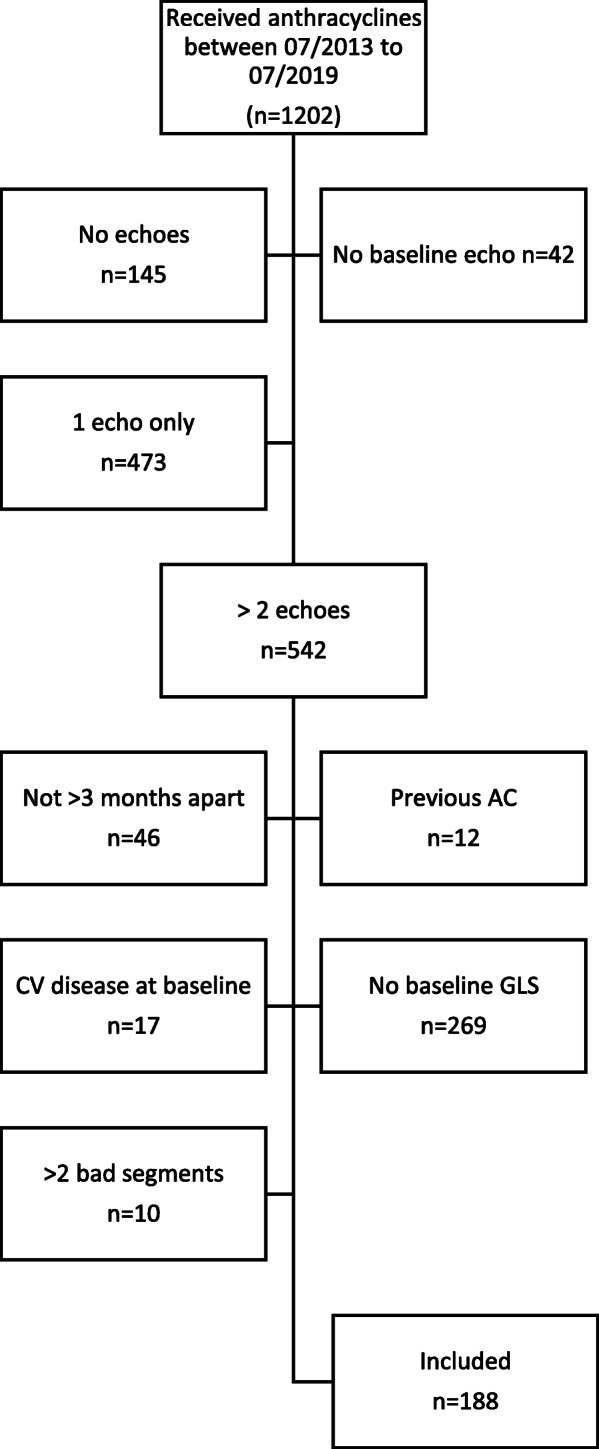
Patient Selection Flow Chart

Patients who had a baseline STE within 3 months before the initiation of anthracycline therapy and at least one follow-up echocardiogram 3 months after start of treatment from July 1, 2013, and July 1, 2019 were identified. Patients with a suboptimal strain study (impaired regional tracking in > 2 myocardial segments), an abnormal left ventricular ejection fraction (LVEF) <50%, left bundle branch block, history of coronary artery bypass grafting (CABG), premature ventricular contractions, or a systolic blood pressure > 180 mmHg or diastolic blood pressure > 100 mmHg before treatment were excluded (Fig. 1). Each chart was accessed for baseline demographics (including age, vitals, cardiovascular (CV) risk factors, medical comorbidities), echocardiography studies, and clinical outcomes (symptomatic heart failure, CV mortality, and non-CV death).

Pre-existing cardiovascular risk factors and medical comorbidities extracted from the electronic medical records, included: body mass index (BMI), tobacco use, diabetes, hypertension, hyperlipidemia, coronary artery disease, hypothyroidism, and a family history of atherosclerotic cardiovascular disease. Cumulative anthracycline dose was calculated based on the following rapid infusion doxorubicin CTRD equivalence: epirubicin, 0.66; daunorubicin, 0.75; and idarubicin, 0.53 [10, 11].

### Echocardiography strain protocol and endpoints

The primary endpoint of the study was CTRD, defined as an LVEF <50% and absolute decline > 10% in LVEF from baseline echocardiography. LVEF was calculated using quantitative (modified Simpson’s biplane method) and/or visual analysis of LVEF according to the American Society of Echocardiography standards [12].

The vendors utilized in the strain analysis included the most recent upgraded versions of GE EchoPAC and TomTec Image-Arena. The endocardial borders were traced from 3 apical viewers. Global longitudinal strain was calculated by measuring the entire endocardial line length at the end-diastole and end-systole in each view and averaging the results from the 3 views. Each measurement was taken from the average of 3 consecutive cardiac cycles. The strain quality was assessed objectively by a second observer as bad if more than two segments were un-visualized.

Secondary end points of all-cause mortality and CV mortality were assessed between the patients with normal and decreased baseline GLS. Decreased baseline GLS was defined as ≥-18% [13].

### Statistical analysis

Baseline characteristics were summarized according to CTRD status and baseline GLS status (abnormal vs normal). All data were presented as mean ± SD for continuous variables and number and % for categorical variables. Chi-square or Fisher’s exact test for categorical variables and t-test or Mann-Whitney test for continuous variables were used to determine association of pre-treatment variables with decreased baseline GLS and CTRD. The normality assumption was tested by the Shapiro –Wilk tests.

Kaplan-Meier survival curves for overall mortality was calculated in strata defined by decreased vs. normal GLS. Time-on-study was used as time scale in all survival analyses: Time begins at first anthracycline dose and ends in date of death or date of last follow-up. Two-sided log rank tests defined significance. The overall survival at 1-year, 3-year, and 5-year intervals between decreased and normal GLS were compared with the pseudo-value approach [12]. Cumulative incidence of cardiovascular related mortality was calculated after adjusted for the competing risk of non-cardiovascular death. Univariable and multivariable Cox proportional hazards regression models were used to examine associations between demographics and clinical variables with CTRD. The multivariable model included baseline GLS and variables selected from backward elimination with the significance level for removal from the model of 0.05. The full model included all statistically significant variables with *p*-value < 0.05 in the univariable models. Subsequently, the same multivariable analysis was done when GLS modeled as a categorical variable (decreased vs. normal). The Cox proportionality assumption was verified by including time-dependent interactions of covariates with survival time in the models. There were no violations of this assumption in all models. The Hosmer and Lemeshow type goodness-of-fit statistics for the Cox proportional hazards model was used to check the model fitting [14].

All analyses were performed with STATA version 16 (StataCorp. 2019. Stata Statistical Software: Release 16. College Station, TX: StataCorp LLC). Statistical significance was defined as two-tailed *p* < 0.05 for all tests.

## Results

### CTRD vs normal

A total of 188 patients were included in our study. Twenty-three patients (12.23%) had CTRD during their treatment course. Baseline characteristics are presented by CTRD in Table 1. The mean baseline GLS of patients who experienced *cardiotoxicity* CTRD during treatment with anthracyclines significantly differed from the mean baseline GLS of patients who did not have CTRD (-17.51% ± 2.77% vs -19.36% ± 2.86%; *p* = 0.004). Patients in the CTRD group were more likely to be male (52.17% vs 30.30%, *p* = 0.056) and were older (60.87 ± 10.89 vs 52.92 ± 14.59, *p* = 0.013). A history of hypertension was more common in the CTRD group (69.57% vs 38.79%, *p* = 0.007) and CTRD patients also had higher baseline systolic blood pressure (137.74 ± 23.86 vs 128.22 ± 16.35, *p* = 0.015). In addition, patients with CTRD were more likely to be treated for a hematologic malignancy (78.26% vs 49.09%, *p* = 0.018). Although within normal values, LVEF was significantly lower in the CTRD group (61.7 ± 4.60 vs 64.43 ± 3.73, *p* = 0.002). Cumulative anthracycline dose was lower in the CTRD group (118.02 mg/m^2^ ± 93.32 mg/m^2^ vs 175.62 mg/m^2^ ± 102.49 mg/m^2^, *p* = 0.013). However, there was no significant difference in the total number of cycles in patients with CTRD vs controls, when grouping by breast cancer vs all other cancers (4.5 vs 3.9, P value 0.25, for breast cancer and 3.3 vs 3.9, P value 0.26, for all other cancers).

**Table 1:** Baseline characteristic by cardiotoxicity (defined as drop EF>10% from baseline and EF< 50% at each time measurement)

	**Total** ***N*** **= 188**	**Cardiotoxicity**		***P*-value**
		**No** ***N*** **= 165**	**Yes** ***N*** **= 23**	
Age (years)	53.89 ± 14.40	52.92 ± 14.59	60.87 ± 10.89	0.013
Male	62 (32.98)	50 (30.3)	12 (52.17)	0.056
BMI (kg/m^2^)	28.40 ± 6.08	28.40 ± 6.20	28.44 ± 5.19	0.98
Race				0.40
Asian	5 (2.66)	5 (3.03)	0 (0.00)	
Black	24 (12.77)	21 (12.73)	3 (13.04)	
Caucasian	99 (52.66)	84 (50.91)	15 (65.22)	
Hispanic	50 (26.60)	47 (28.48)	3 (13.04)	
Other	10 (5.32)	8 (4.85)	2 (8.70)	
Systolic	129.42 ± 17.68	128.22 ± 16.35	137.74 ± 23.86	0.015
Diastolic	70.97 ± 10.66	70.67 ± 10.62	73.09 ± 10.93	0.31
Heart Rate	76.38 ± 13.31	76.23 ± 13.28	77.53 ± 13.85	0.69
Family history of Heart Disease	19 (10.11)	15 (9.09)	4 (17.39)	0.26
Diabetes	35 (18.62)	29 (17.58)	6 (26.09)	0.39
Hypertension	80 (42.55)	64 (38.79)	16 (69.57)	0.007
Hyperlipidemia	52 (27.66)	44 (26.67)	8 (34.78)	0.46
Coronary Artery Disease	9 (4.79)	8 (4.85)	1 (4.35)	1.00
Hypothyroidism	21 (11.17)	20 (12.12)	1 (4.35)	0.48
Smoking	48 (25.53)	33 (20.00)	15 (65.22)	< 0.001
Ejection Fraction	64.09 ± 3.94	64.43 ± 3.73	61.70 ± 4.60	0.002
Baseline GLS	-19.13 ± 2.91	-19.36 ± 2.86	-17.51 ± 2.77	0.004
Cancer type				0.018
Breast	80 (42.55)	76 (46.06)	4 (17.39)	
Hematologic	99 (52.66)	81 (49.09)	18 (78.26)	
Other	9 (4.79)	8 (4.85)	1 (4.35)	
Chemotherapy dose	168.70 ± 102.91	175.62 ± 102.49	118.02 ± 93.32	0.013

Data were presented as mean ± SD for continuous variables and number (%) for categorical variables. Chi-square or Fisher’s exact test (categorical variables) and t-test or Mann-Whitney test (continuous variables) were used to compare patients between cardiotoxicity status

### Decreased versus normal baseline GLS

Patients with an decreased baseline GLS were most likely male (43.94% vs 27.05%, *p* = 0.023), had a higher BMI (30.03 ± 5.99 vs 27.53 ± 5.96, *p* = 0.007), and had a lower baseline LVEF (62.39% ± 4.43% vs. 65.01% ± 3.32%, *p* < 0.001). There were no differences in age, comorbidities, baseline blood pressure and heart rate (by echocardiogram report) or cancer type (Table 2).

**Table 2:** Baseline characteristic by baseline GLS [Decreased GLS defined as GLS≥(-18%)]

	**Total** ***N*** **= 188**	**Decreased Baseline GLS**		***P*-value**
		**No** ***N*** **= 122**	**Yes** ***N*** **= 66**	
Age (years)	53.89 ± 14.40	53.62 ± 14.86	54.38 ± 13.60	0.73
Male	62 (32.98)	33 (27.05)	29 (43.94)	0.023
BMI (kg/m^2^)	28.40 ± 6.08	27.53 ± 5.96	30.03 ± 5.99	0.007
Race				0.91
Asian	5 (2.66)	4 (3.28)	1 (1.52)	
Black	24 (12.77)	14 (11.48)	10 (15.15)	
Caucasian	99 (52.66)	65 (53.28)	34 (51.52)	
Hispanic	50 (26.60)	33 (27.05)	17 (25.76)	
Other	10 (5.32)	6 (4.92)	4 (6.06)	
Systolic	129.42 ± 17.68	127.97 ± 16.42	132.17 ± 19.69	0.13
Diastolic	70.97 ± 10.66	69.97 ± 9.84	72.86 ± 11.91	0.083
Heart Rate	76.38 ± 13.31	76.06 ± 12.80	76.95 ± 14.27	0.68
Family History of Heart Disease	19 (10.11)	13 (10.66)	6 (9.09)	0.81
Diabetes	35 (18.62)	19 (15.57)	16 (24.24)	0.17
Hypertension	80 (42.55)	50 (40.98)	30 (45.45)	0.64
Hyperlipidemia	52 (27.66)	35 (28.69)	17 (25.76)	0.73
Coronary Artery Disease	9 (4.79)	4 (3.28)	5 (7.58)	0.28
Hypothyroidism	21 (11.17)	16 (13.11)	5 (7.58)	0.33
Smoking	48 (25.53)	27 (22.13)	21 (31.82)	0.16
Ejection Fraction	64.09 ± 3.94	65.01 ± 3.32	62.39 ± 4.43	< 0.001
Cancer type				0.24
Breast	80 (42.55)	57 (46.72)	23 (34.85)	
Hematologic	99 (52.66)	60 (49.18)	39 (59.09)	
Other	9 (4.79)	5 (4.10)	4 (6.06)	
Chemotherapy dose	168.70 ± 102.91	165.56 ± 95.49	174.27 ± 115.47	0.58

Data were presented as mean ± SD for continuous variables and number (%) for categorical variables. Chi-square or Fisher’s exact test (categorical variables) and t-test or Mann-Whitney test (continuous variables) were used to compare patients between abnormal and normal GLS

### Univariable and multivariable analyses

Median time to > 10% reduction in LVEF was 107 days (range 12.5-729). In a univariable Cox proportional hazards regression analysis, baseline GLS as continuous variable or categorical variable was significantly associated with CTRD (HR = 1.20, 95% CI: 1.05, 1.38; *p* = 0.01 for continuous and HR = 4.44, 95% CI: 1.83, 10.8; *p* < 0.001 as categorical). In addition to GLS, age, male gender, baseline systolic pressure, baseline LVEF, history of hypertension and tobacco use, breast cancer, and cumulative chemotherapy dose were found significant in the univariable model for CTRD. In the multivariable analysis, baseline GLS as continuous variable or decreased GLS (≥(-18%)), baseline systolic pressure, history of tobacco use, and chemotherapy dose were significant (Table 3). The final models fitted well (the p-values for the Hosmer-Lemeshow type goodness-of-fit from likelihood-ratio test were *p* = 0.79 when GLS as continuous variable and *p* = 0.51 when GLS as categorical variable).

**Table 3:** Hazards ratio (HR) and 95% CI for developing cardiotoxicity

	Univariable		Multivariable	
	HR (95% CI)	*p*-value	HR (95% CI)	*p*-value
GLS	1.20 (1.05,1.38)	0.01	1.17 (1.00,1.36)	0.044
GLS[≥(-18%) vs <(-18%)]	4.44 (1.83,10.8)	< 0.001	3.54 (1.34,9.35)	0.011
Age	1.04 (1.01,1.07)	0.01		
Male	2.56 (1.13,5.8)	0.02		
BMI	1.01 (.94,1.08)	0.87		
Systolic	1.03 (1,1.05)	0.02	1.03 (1.01,1.05)	0.012
HR	1.01 (.97,1.04)	0.7		
LVEF	0.85 (.76,.94)	< 0.001		
Caucasian	1.67 (.71,3.94)	0.24		
Family Hx of heart disease	1.99 (.68,5.86)	0.21		
Diabetes	1.76 (.69,4.49)	0.23		
HTN	3.55 (1.46,8.63)	0.01		
Hyperlipidemia	1.51 (.64,3.56)	0.35		
CAD	1.03 (.14,7.72)	0.97		
Hypothyroidism	0.34 (.05,2.53)	0.29		
Smoking	6.86 (2.9,16.21)	< 0.001	5.29 (2.09,13.39)	< 0.001
Breast cancer	0.24 (.08,.69)	0.01		
Chemotherapy dose	0.99 (.99,1)	0.01	0.994 (0.989,0.999)	0.018

Multivariable analysis included GLS and variables selected from stepwise selection procedure. The full model included all variables with *p*-value< 0.05 from univariable analysis. The HR (95% CI) were presented when GLS was modeled as continuous variable. Then the same multivariable analysis was done when GLS modeled as a categorical variable [defined as ≥(-18%) vs <(-18%)]

The optimal cutoff point for GLS at baseline with CTRD was -18.05%. The sensitivity was 0.70 and specificity was 0.70. The area under ROC curve was 0.70.

**Fig. 2:**
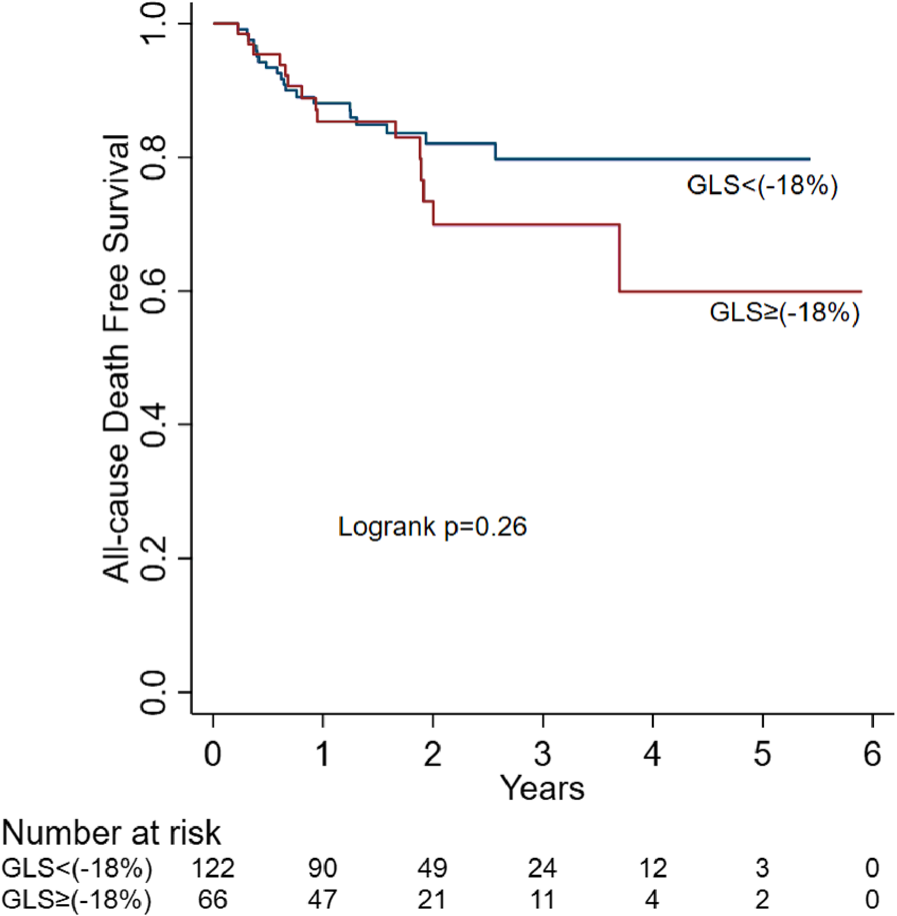
KM plot for all-cause mortality

**Fig. 3:**
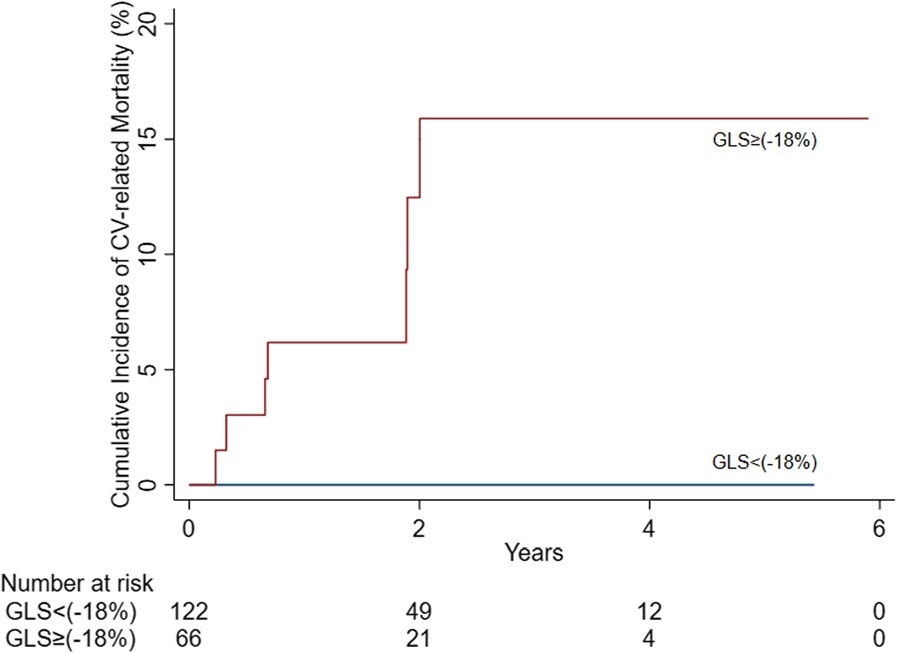
Cumulative incidence of CV-related mortality adjusted for competing risk of non-cardiovascular death

There was no significant difference in overall all-cause mortality between patients with decreased vs. normal baseline GLS strain (log-rank *p* = 0.26). The p values for the survival comparison between GLS≥ (-18%) vs GLS< (-18%) are 0.67 at 1 year, 0.33 at 3 years and 0.13 at 5 years, respectively (Fig. 2, Table 4). However, CV-related mortality was significantly higher in the decreased GLS>-18% group (Log-rank *p* < 0.001) (Fig. 3).

**Table 4:** Survival probability at different time point by GLS

	GLS<(-18%)	GLS≥(-18%)	*P*-value
1 year	0.87 (0.80 – 0.92)	0.85 (0.74 – 0.92)	0.67
3 year	0.79 (0.69 – 0.86)	0.71 (0.54 – 0.83)	0.33
5 year	0.79 (0.69 – 0.86)	0.62 (0.39 – 0.79)	0.13

## Discussion

In this study, we found that patients with normal LVEF and decreased baseline GLS were more likely not only to develop CTRD, defined by a drop of EF > 10% points and to final value <50%, but these patients with baseline GLS abnormalities also had a statistically significant increase in CV-related mortality. In fact, a baseline GLS>-18% was associated with a greater than 4-fold increased risk of CTRD. Furthermore, there was an increased risk of CTRD in the group with an abnormal baseline GLS despite a lower cumulative anthracycline dose in this group compared to controls.

The American Society of Clinical Oncology (ASCO) recommends heightened monitoring for patients at higher risk of CTRD, and these risk factors include higher dose, concomitant her-2 antagonists or chest radiation, age, traditional cardiac risk factors, and prior myocardial infarction. In addition, compromised cardiac function by imaging --specifically valvular disease or LVEF < 55%-- are considered risk factors. The use of baseline strain assessment by itself as a risk assessment (as opposed to its measurement only to establish a baseline to measure subsequent relative reduction in GLS) is not currently in the guidelines and our study brings additional awareness to emerging data that shows that consideration should be given to the inclusion of baseline GLS as risk factor by itself.

Beyond the established risk factors including cumulative anthracycline dose, it is unclear why some patients without these risk factors develop CTRD. There is recent preliminary evidence that some of these patients may be genetically predisposed. A recent study of 213 chemotherapy cardiomyopathy patients from several cohorts found 26 (12%) had genetic variants, predominantly truncating variants in the titin gene (TTN), that are known to cause familial dilated cardiomyopathy. Furthermore, a mouse model with TTN mutations only developed heart failure if they were also treated with anthracyclines while wild type mice treated with the same dose did not develop heart failure. This reinforces the idea that some patients may be predisposed to develop cardiomyopathy and that chemotherapy is the “second hit” that causes cardiomyopathy. Similarly, baseline abnormalities in strain may reflect a structurally abnormal heart that is more susceptible to chemotherapy.

Changes in GLS in patients while receiving anthracycline-based chemotherapy is a well-established predictor of CTRD. The role of baseline GLS is certainly less established, but other studies have also found that it may predict CTRD. Our study has two important distinguishing features from the prior studies. First, most of these studies have only been in patients with hematological malignancies, which have a higher upfront anthracycline exposure. This is the first study to date to our knowledge evaluating decreased baseline GLS in a broad cohort of cancer patients with lower pre-treatment risk of developing anthracycline CTRD per ASCO. Patients with hematological malignancies were more likely to have CTRD than breast cancer patients. The second notable difference is that our study is the only, to our knowledge, to exclude patients with borderline LVEF. Our study did not include anyone with an EF < 55%, thus affirming that decreased baseline GLS has clinical utility in predicting anthracycline CTRD in patients with normal LVEF. One study found an abnormal baseline GLS (cutoff defined as >-15) to be the highest predictive factor in their risk score of patients with acute leukemia. Importantly, 30 of the 450 patients in this study had an LVEF < 53% at baseline and this characteristic was included in their risk score [9].

In addition, decreased GLS was associated with an increase in CV mortality. This reinforces findings from other anthracycline studies. In a study of 450 patients with hematological malignancies, baseline GLS >-17.5 was associated with a combination of cardiac death or symptomatic heart failure [7]. Although confounded by inclusion of patients with a borderline LVEF 50-55%, Kang et al. found that a GLS> -15 was associated with all-cause mortality [9]. Currently, abnormal GLS in and of itself is not established in guidelines to represent Stage B heart failure, although there is evidence and debate in favor of the inclusion [15, 16]. Our study adds additional support that decreased GLS represents may represent microstructural changes in myocardium with significant prognostic implications.

In addition to highlighting the increased risk associated with abnormal baseline GLS abnormalities, it remains unknown if that risk can be mitigated by cardioprotective strategies, Recently, for the first time, the use of strain to guide clinical decision making has been studied, demonstrating that using cardioprotective medications for patients with a change in strain (12% relative reduction) during chemotherapy can reduce the incidence of CTRD. Future studies should investigate cardioprotective strategies in patients with baseline GLS abnormalities [17].

## Study limitations

First, this is a single center study that was performed retrospectively. Another limitation is that even though patients with an LVEF < 55% were excluded from the study, there was a small but statistically significant lower baseline LVEF in the CTRD group. However, decreased baseline GLS was more predictive than LVEF in predicting CTRD, reinforcing the concept that baseline GLS has additional utility in identifying patients at low pre-treatment risk of anthracycline CTRD. Finally, there were important baseline differences and thus potential confounding factors between the groups, including higher blood pressure, history of hypertension, increased age and more prevalent smoking history in the CTRD group. Regardless, baseline GLS was more predictive of each of these factors in the multivariate analysis.

## Conclusion

Baseline GLS has predictive value in identifying patients with low-to-moderate pre-treatment risk of anthracycline CTRD. Furthermore, decreased baseline GLS may be associated with increased CV mortality among patients receiving anthracyclines. The results of this study favor broader implementation of STE in cardio-oncology practice for thorough risk stratification and identification of patients at higher risk for developing anthracycline-induced CTRD than indicated by pre-treatment comorbidities and cancer therapy related risk factors.

## Data Availability

The datasets used and/or analyzed during the current study are available from the corresponding author on reasonable request.

## Abbreviations

STE: 2-dimensional speckle tracking strain protocol echocardiography
BMI: Body mass index
CABG: Coronary artery bypass grafting
GLS: Global longitudinal strain
LVEF: Left ventricular ejection fraction
